# Efficacy and safety of inhaled calcium lactate PUR118 in the ozone challenge model - a clinical trial

**DOI:** 10.1186/s40360-015-0021-1

**Published:** 2015-08-12

**Authors:** Olaf Holz, H. Biller, M. Mueller, K. Kane, M. Rosano, J. Hanrahan, D. L. Hava, J. M. Hohlfeld

**Affiliations:** Department of Clinical Airway Research, Fraunhofer Institute for Toxicology and Experimental Medicine, 30625 Hannover, Germany; Member of the German Center for Lung Research (BREATH), Hannover, Germany; Pulmatrix, Inc., Lexington, MA USA

## Abstract

**Background:**

The ozone challenge model can be used to assess the efficacy of anti-inflammatory compounds in early phases of clinical drug development. PUR118, a calcium salt based formulation engineered in the iSPERSE^TM^ dry powder delivery technology, is a novel anti-inflammatory drug for COPD. Here we evaluated the efficacy and safety of three doses of PUR118 in attenuating ozone-induced airway inflammation in healthy volunteers.

**Methods:**

In a single-blind, phase 1B proof of concept study, 24 subjects were enrolled to sequentially receive three doses of PUR118 (5.5 mg, *n* = 18; 11.0 mg, *n* = 18; 2.8 mg, *n* = 16). Each dose was inhaled 3 times (1, 13, 25 h, preceded by 2 puffs salbutamol) before the ozone exposure (250 ppb, 3 h intermittent exercise). Sputum was induced 3 h after the end of exposure.

**Results:**

Sputum neutrophils, sputum CD14+ cells, as well as concentrations of IL1B, IL6, IL8, MMP9, and TNFA in sputum supernatant significantly increased after ozone exposure (*n* = 24). The percentage of sputum neutrophils (*n* = 12 who completed all treatments) did not change following treatment with different doses of PUR118. The high dose treatment group (*n* = 16) showed a decrease in the percentage and number of sputum macrophages (*p* ≤ 0.05) as well as a decrease in blood neutrophils (*p* = 0.04), and an increase in blood CD14 + cells (*p* = 0.04) compared to baseline. All dosages of PUR118 were safe and well tolerated.

**Conclusion:**

Ozone challenge resulted in the expected and significant increase of sputum inflammatory parameters. Treatment with multiple rising doses of PUR118 was safe and three applications within 25 h prior to the ozone challenge had small effects on ozone-induced airway inflammation.

**Trial registration:**

ClinicalTrials.gov: NCT01690949. Registered 12 September 2012.

**Electronic supplementary material:**

The online version of this article (doi:10.1186/s40360-015-0021-1) contains supplementary material, which is available to authorized users.

## Background

Chronic obstructive pulmonary disease (COPD) is a major health problem due to the increasing prevalence with chronic morbidity and potential mortality of the disease [1]. On the individual level, the disease is characterized by dyspnea on exertion, chronic cough and sputum production, partially reversible airway obstruction and a variety of systemic comorbidities [2]. The underlying pathophysiology is often driven by cigarette-smoking resulting in a chronic inflammation of the airways which poorly responds to anti-inflammatory therapy [3]. In particular and in contrast to asthma, corticosteroids have only little clinical benefit in the treatment of COPD [1]. Therefore, the development of novel drugs with anti-inflammatory properties seems warranted.

Numerous anti-inflammatory approaches targeting neutrophil and monocyte function and recruitment have been developed and tested clinically over the last decade, including p38 MAP-kinase inhibitors, CXCR2 antagonists, 5-lipoxygenase inhibitors, tumor necrosis factor alpha (TNFA), Interleukin (IL)1 or IL17 specific antibodies [4], but none have been approved. Roflumilast a licensed oral drug for COPD has anti-inflammatory activity in man [5, 6], but the clinical benefit is limited to a subtype of patients with productive cough and frequent exacerbations. While many of the above mentioned investigational new drugs target very specific receptors or pathways with relevance in COPD, anti-inflammatory strategies with a broad mode of action are scarce. A novel concept to provide anti-inflammatory and airway protective effects is the inhaled delivery of calcium ions to the airways [7, 8]. PUR118 is a calcium salt based formulation engineered in the iSPERSE^TM^ dry powder delivery technology. The active moiety of PUR118 is a calcium ion and formulated as calcium lactate inhalation powder (CLIP) that can be administered by a capsule based dry powder inhaler. PUR118 was developed to act on the ciliated epithelium in the conducting airways and its efficacy is driven jointly by the biophysical impact on the airway surface liquid and its alteration of host responses in the epithelium. Preclinically, calcium based formulations have been shown to alter the viscoelastic properties of airway surface liquid, enhance the barrier properties of airway mucins and enhance mucociliary clearance [9–11]. In animal models, CLIP has demonstrated prophylactic and therapeutic efficacy in multiple models of respiratory infection and have shown to reduce the severity of airway inflammation in *in vivo* models of chronic respiratory diseases [7, 8, 11]. The broad and multiple effects of PUR118 may be expected to result in clinical impact on exacerbation rates in COPD and cystic fibrosis.

The ozone challenge model has been shown to be useful for proof of concept studies in early drug development of novel compounds targeting neutrophilic airway inflammation [12–15]. In this model healthy volunteer subjects are exposed to ozone for 3 h under intermittent exercise, which results in a transient, reproducible increase in sputum neutrophils as well as sputum biomarkers such as IL8 or myeloperoxidase (MPO), inflammatory features also observed in COPD. The recently updated German medication law (AMG) now requires a manufacturing license for ozone, which has been granted for the Fraunhofer ozone exposure chamber in 2012, following a comprehensive validation process.

It was the aim of this proof-of-concept study to test whether the protective effect on airway epithelium of PUR118 can modulate ozone-induced airway inflammation and to investigate the safety of multiple ascending doses of PUR118 in healthy non-smoking adult volunteers.

## Methods

### Study design

**Fig. 1:**
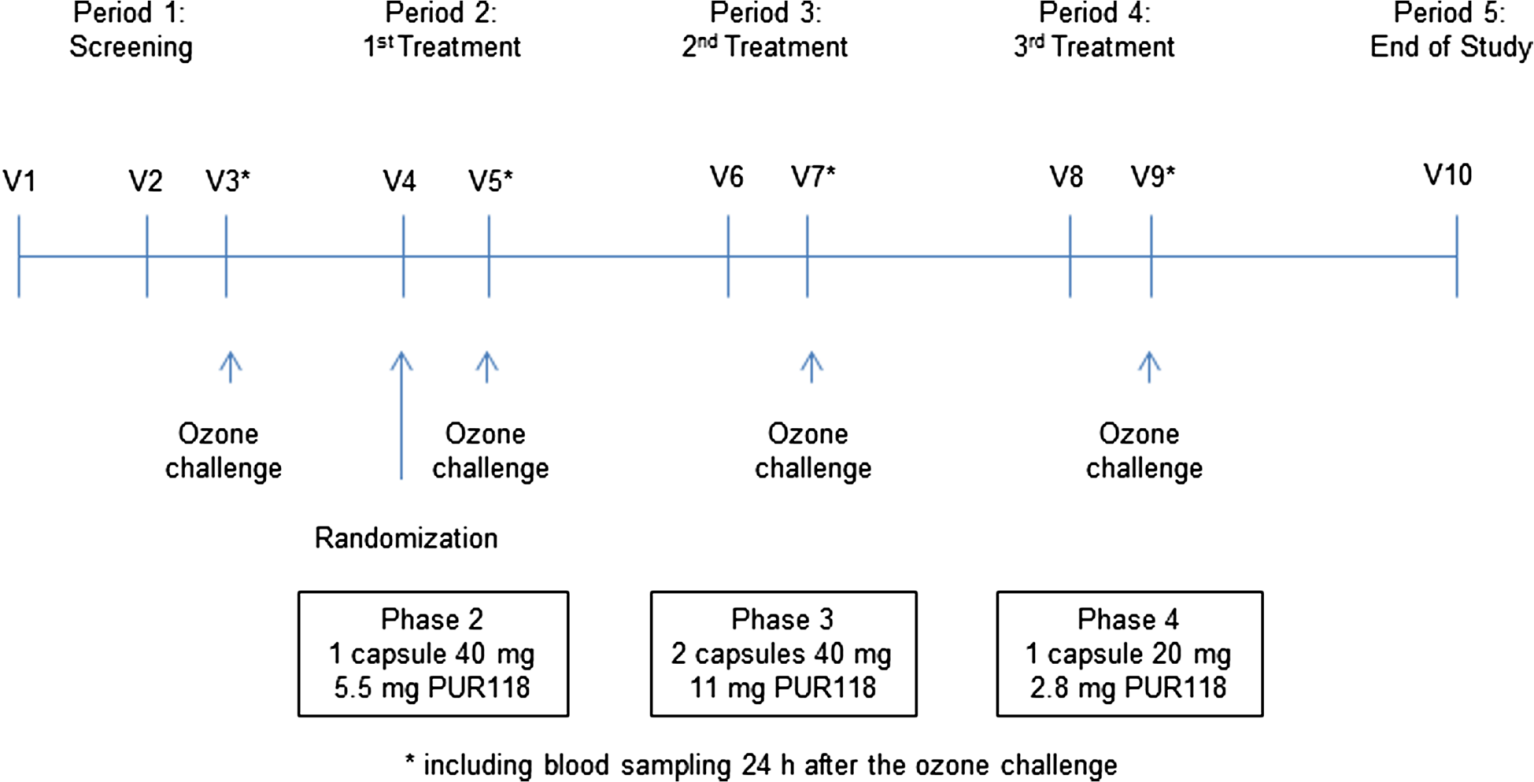
Study design. After randomization subjects were treated with 3 different doses of PUR118 in the displayed sequence (except for 1 subject, who inhaled in the sequence high, medium low dose)

The study was conducted as a single-blind evaluation of PUR118 in five periods separated by at least 2 weeks ‘wash-out’ to allow the ozone-induced airway inflammation to subside (Fig. 1). In period 1, healthy volunteers signed the informed consent, were screened for inclusion and exclusion criteria and performed the baseline ozone challenge. At visit 1, a physical examination, electrocardiogram (ECG), and a spirometry were performed, and the medical history, use of concomitant medications, vital signs, height, and weight were recorded. Blood was collected for clinical laboratory evaluations, and sputum was induced to determine the ability of subjects to produce sufficient quantity for evaluation. Qualified subjects returned within a week for a qualifying ozone challenge over 2 days (visit 2 and 3), that also served as baseline (BL) challenge (salbutamol treatment prior to challenge only, no PUR118 medication on visit 2). Spirometry was checked hourly during the ozone exposure as well as 6 h and 24 h after the start of ozone challenge. Blood samples were collected pre-dose and 75 min post salbutamol (no PUR118 treatment at BL) and 7 and 24 h post ozone inhalation. A sputum sample was induced 6 h post-ozone. Volunteers were included in the study, if a >10 % increase in the absolute percentage of sputum neutrophils was observed in response to ozone.

In periods 2, 3, and 4 qualified subjects returned for two visits over two consecutive days per period. At visit 4, 6, and 8 vital signs were assessed, and changes in concomitant medications and the occurrence of adverse events were documented. Volunteers inhaled their first dose of study medication during the visit according to the sequence shown in Fig. 1. Vital signs and spirometry were recorded for up to 1 h post dose and a blood sample for evaluation of electrolytes was collected 1 h after the end of dosing. Subjects administered the second dose of PUR118 at home approximately 12 h after the first dose. At visit 5, 7, and 9, the day after the first PUR118 dose, the third dose of study medication was administered following pre-dose procedures as described above. The ozone exposure started 1 h post study drug administration at visit 5, 7 and 9. Procedures during and after ozone exposure were described above and were identical to the baseline ozone exposure. A follow-up visit was performed 2 weeks after visit 9 (period 5) to perform a final safety evaluation including a physical examination, vital signs, ECG, spirometry, and collection of a blood sample for clinical laboratory assessments.

### Subject eligibility criteria

Twenty-four healthy, non-smoking subjects were included into the study and for the safety analysis data set (Table 1).

**Table 1:** Subject demographics (*n* = 24)

Male	n (%)	12	(50.0)
Female	n (%)	12	(50.0)
Age (years)	Median (range)	35	(20–48)
Body height (cm)	Median (range)	175.5	(158.0–194.0)
Body weight (kg)	Median (range)	75.5	(57.0–110.0)
BMI (kg/m^2^)	Median (range)	24.4	(19.6–29.8)

*BMI* body mass index, *n* number of subjects

The main inclusion criteria were: 1. Healthy males or non pregnant, non lactating healthy females age 18–50 years; 2. Body Mass Index (BMI) of 18–35 kg/m^2^ or, if outside the range, considered not clinically significant by the Investigator; 3. Must be willing/able to give informed consent and adhere to protocol schedule and restrictions; 4. Females of child-bearing potential must have negative pregnancy test and agree to use two methods of birth control throughout the study; 5. Males must agree to use an acceptable method of birth control throughout the study; 6. Must be able to produce acceptable sputum sample by induction; 7. Must respond to ozone inhalation with a >10 % increase in the absolute percentage of sputum neutrophils and the total neutrophils (neutrophils/g sputum) must increase by at least 50 % from the sputum neutrophil count at screening; 8. Volunteer is a non-smokers or ex-smoker of at least 12 months’ duration prior to screening with a history of less than 1 pack year.

The main exclusion criteria were: 1. Regular alcohol consumption in males >21 units per week and females >14 units per week (1 unit = ½ pint beer, 25 mL of 40 % spirit or a 125 mL glass of wine); 2. Positive drugs of abuse test, urine cotinine test, or alcohol breath test; 3. Positive HBV, HCV or HIV results; 4. Serious adverse reaction or serious hypersensitivity to any drug or the investigational medicinal product, or salbutamol; 5. Volunteers receiving chronic medication other than oral contraceptives; 6. Screening forced expiratory volume in 1 s (FEV1) is <80 % of the predicted value for their age, gender, height and race and/or their FEV1/forced vital capacity (FVC) ratio is below 70 %; 7. Volunteers with significant occupational exposure to respiratory irritants or toxins; 8. Upper respiratory tract infection within 30 days of the first study day, or lower respiratory tract infection within the last 3 months; 9. Volunteers taking any medication that may affect the respiratory tract within 30 days of the first study day; 10. Volunteers with a history of asthma; 11. Participation in a clinical research study within the previous 3 months.

### Ethics statement and informed consent

The study was conducted in accordance with Good Clinical Practice and the Declaration of Helsinki. Subjects gave their written informed consent. The study was approved by the Ethical Committee of Hannover Medical School (Statement No. 6148 M) and the competent authority (Bundesinstitut für Arzneimittel und Medizinprodukte, BfArM) and registered at Clinical trials.gov (NCT01690949). The study site was the Fraunhofer Institute for Toxicology and Experimental Medicine in Hannover, Germany.  The CONSORT checklist can be found as Additional file 1: Doc 1. The first patient was included on June 25^th^ 2012 and the last patients follow up was on 30^th^ April 2013. For more information refer to (Additional file 2: Doc 2).

### Treatment

Unit doses of PUR118 were provided in pre-metered size 3 hydroxy-propyl-methylcellulose (HPMC) clear capsules and administered according to Table 2 using a reloadable, passive dry powder inhaler (DPI, Monodose Inhaler RS01 Model 7 high-resistance, Plastiape, Italy). For safety reasons the administration of pre-dose bronchodilators is common when administering high dose dry powder formulations, therefore each dose of PUR118 was preceded by 2 puffs of salbutamol (200 μg in total) 15–30 min prior to the inhalation of PUR118. The baseline evaluation was performed after inhalation of 200 μg salbutamol only (no other treatment). The subjects were blinded with respect to the dose of treatment.

**Table 2:** Dosage

Baseline	*n* = 24	200 μg salbutamol	Untreated
Low dose	*n* = 16	200 μg salbutamol	2.8 mg PUR118 (1 dose every 12 h for 36 h)
Mid dose	*n* = 18	200 μg salbutamol	5.5 mg PUR118 (1 dose every 12 h for 36 h)
High dose	*n* = 18	200 μg salbutamol	11 mg PUR118 (1 dose every 12 h for 36 h)

### Exposure to ozone

Subjects were exposed to 250 ppb ozone (O3) in the Fraunhofer-ITEM exposure chamber under intermittent bicycle ergometer exercise (15 min exercise alternating with 15 min rest, ventilation rate during exercise adjusted to 20 l/min/m^2^ body surface). During exposure, heart rate was monitored continuously via ECG, and blood pressure was measured every 30 min. The exposure chamber (2.7 × 2.3 × 2.5 m^3^) was ventilated by conditioned air with temperature of 20–25 °C and relative humidity of 40–60 %. High purity ozone input was generated by the method of surface discharge using a COM ADM generator (ANSEROS GmbH, Tübingen), which was operated with 100 % medical oxygen. Ozone concentration in the exposure chamber was continuously controlled via an ozone analyzer and controlling unit (Ozomat MP, ANSEROS, Tübingen). Safety of exposures was ensured by built-in maximum limits of ozone flux as well as by an independent, monitor-type ozone analyser (400A, MLU-Messtechnik für Luft und Umwelt GmbH, Essen) working by the principle of UV absorption. This instrument was used to monitor the target concentration of 250 ppb in the exposure chamber continuously and was routinely checked by official authorities (TÜV Nord Umweltschutz GmbH) using a certified instrument.

### Analysis of efficacy and safety endpoints

Changes in sputum total cell count, differential cell count including monocytes by flow cytometry, and sputum supernatant concentration of interleukin (IL) 8, matrix metalloproteinase (MMP) 9, IL6, and IL1B were evaluated between baseline and ozone exposure (6 h post-initiation of ozone challenge). Changes in CD14+ monocytes, blood polymorphonuclear neutrophils and their level of activation (CD11b + neutrophils), as well as changes in blood C-reactive protein (CRP) and CC16 (Clara cell protein16kDa, SCGB1A1) from pre-ozone challenge to 7 and 24 h post-initiation of -ozone challenge were also analyzed. Blood was drawn by venous puncture to separate serum which was stored at −80 °C prior to the analysis. Spirometry was used to assess the changes of FEV_1_ and FVC from pre-dose to all post-dose time points. Furthermore, we documented all clinical signs, symptoms, clinical safety laboratory tests, vital signs, electrocardiogram (ECG), oxygen saturation and adverse events (AEs) including serious adverse events (SAEs).

### Induced sputum

Sputum induction was performed as previously described [16]. Briefly, subjects inhaled 3, 4 and 5 % hypertonic saline in 3 consecutive 10 min inhalation periods. After each period subjects were asked to produce sputum. Sputum plugs were selected, pooled and homogenized with Sputolysin® (Calbiochem, Darmstadt, Germany). After centrifugation, supernatants were collected and stored at −80 °C before the analysis of biomarkers. After filtration, cytospins were prepared and a differential cell count was performed by counting at least 400 non-squamous cells.

### Flow cytometry of sputum cells

An aliquot of the sputum sample was used for flow-cytometric analysis (Cytomics FC500; Beckman Coulter, Krefeld, Germany) [17]. Staining included fluorochrome-labeled antibodies from Beckman Coulter (CD14 (APC)) and Invitrogen (CD11b-FITC) and the respective non-specific isotype control antibodies from the same sources. To quantify sputum cell subpopulations, leukocytes were differentiated from cellular debris and squamous epithelial cells and further differentiated into leukocyte subpopulations by gating strategies based on light scatter properties (forward scatter: FSc, sideward scatter: SSc) and specific surface markers. To assess the expression of selected cell surface molecules the mean fluorescence intensity (MFI) was measured. Specific isotype controls were subtracted from the respective MFI values.

### Flow cytometry of peripheral blood

Total cell count was performed by AC-T8 hematology analyser (Beckman Coulter) and by morphological gating using flow cytometry. One aliquot of blood was used to measure CD11b + neutrophils and CD14+ monocytes. Briefly, the blood was incubated with goat serum (20 min, 4 °C) and washed prior to incubation with the antibodies and respective isotype controls for 30 min at 4 °C (CD11b-FITC, IsoIgG1-FITC (Invitrogen, Darmstadt, Germany), CD14-PC5 (Beckmann Coulter, Krefeld, Germany)). After washing, cells were lysed (VersaLyse-Reagenz, Beckman Coulter). After two further washing steps samples were stored on ice until analysis by a Beckman Coulter Cytomics FC500 flow cytometer.

### Statistics

Statistical comparisons of each PUR118 dose vs. salbutamol-only treatment (Baseline ozone exposure) were computed using a paired sample t-test. The data distribution was checked before performing such statistical comparison. Data were log transformed if not normally distributed. The 95 % confidence intervals for paired samples on absolute and percentage change were calculated using the t-distribution. An analysis of variance (ANOVA) for repeated measurements was conducted to test for monotone treatment effects.

For all tests, a 2-sided significance level of 5 % was applied, unless otherwise noted. Due to the exploratory nature of this study, no adjustment for multiple testing was done.

### Sample size and power

We aimed to include a minimum of 18 subjects for the analysis. The analysis of the impact of PUR118 on ozone-induced inflammatory cell and neutrophilic airway infiltration in this study was exploratory, so this sample size was largely determined outside of statistical considerations, but is consistent with the number of subjects examined in other ozone challenge clinical trials of this type conducted in the past.

One of the important outcomes relevant to the potential efficacy of PUR was the extent to which PUR118 attenuates ozone-induced neutrophil infiltration into the airways. Based on the experience at the Fraunhofer site, post-challenge neutrophil counts in ozone-responsive healthy volunteers was estimated to be about 60 % (±15 % standard deviation) of total cells 6 h after challenge without prior treatment. Given a mean observed of 15 % (20 %) within-subject variability of ozone neutrophil response, we estimated that at a significance level of α =0.05, the design (18 subjects who complete) would have 99 % (99 %) power to detect a 40 % reduction in neutrophilic airway infiltration, a 99 % (94 %) power to detect a 30 % reduction, and 89 % (67 %) power to identify a 20 % reduction at any of the 3 PUR118 dose levels. As such, a design with a sample size of 18 evaluable subjects was considered appropriate for evaluation of PUR118.

## Results

### Disposition of subjects

**Fig. 2:**
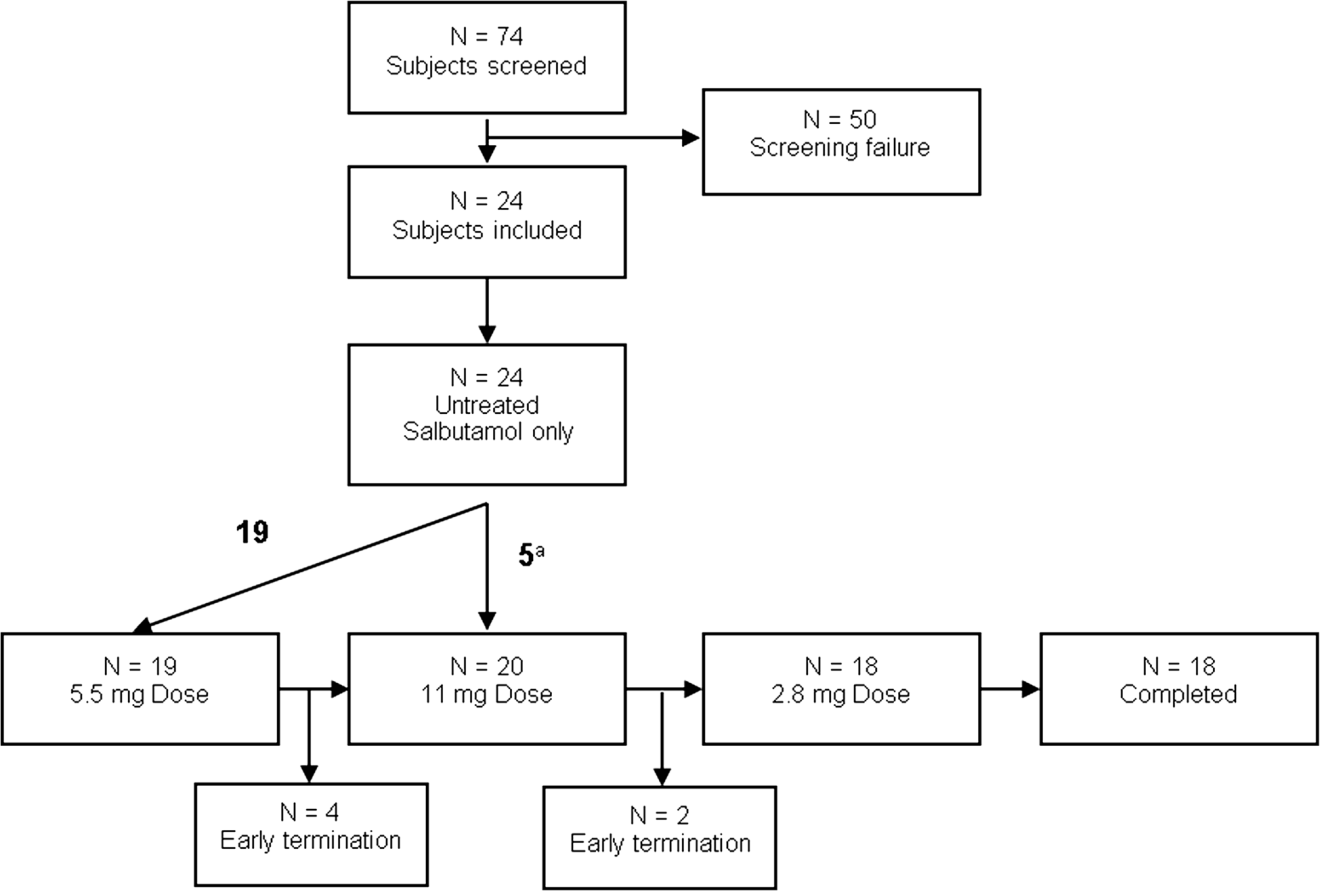
Of the 74 volunteers that were screened 19 subjects were excluded due to insufficient sputum samples. Of these 10 subjects did not produce a sufficient amount of cells to perform all required analysis (<200.000 cells) and 9 subjects showed a percentage of >50 % neutrophils in their sputum, which was chosen as a cut-off point to allow a sufficient increase after ozone exposure. ^a^15 subjects crossed over from the 5.5 mg group, an additional 5 subjects were included directly. *N* = number of subjects

Twenty-four eligible subjects were included into the safety data analysis. Four subjects terminated the study after the first treatment with PUR118 [5.5 mg dose level, (withdrawal of consent of three subjects, decline in pulmonary function during ozone challenge in one subject)] and two subjects terminated the study after the 11 mg dose treatment period due to unrelated AEs (moderate pain in both knees, mild sore throat). Therefore, an additional five subjects were included directly into the 11.0 mg treatment group. A total of 18 subjects completed the study, of which 12 subjects provided complete datasets for treatment with all three doses of PUR118 (Fig. 2). The date of the first subject enrolled was the 25^th^ of June 2012, the date of last subject completed was the 30^th^ April 2013.

### Efficacy

The overall sputum quality in the study was good, the median (interquartile range, IQR) squamous cell contamination being 8.1 (3.0; 15.3) %. In only 3 cases did a subject not produce an adequate sputum sample and the analysis by flow cytometry could not be performed (twice during screening, after 2.8 mg dose treatment).

The effect of ozone exposure on cellular changes from pre- to post-baseline ozone was clearly detectable and is listed in Table 3 (*n* = 24). Post-baseline ozone sputum data measured 6 h post-ozone challenge (i.e. salbutamol only treatment) and the absolute changes from post-baseline ozone challenge during the study for each dose are summarized in (Additional file 3: Table S1). For most parameters only small changes were observed after PUR118 treatment and there was no clear trend that PUR118 altered ozone-induced increase in sputum inflammatory markers. Small reductions of ozone-induced increases in inflammatory markers were observed for some parameters with the low (2.8 mg) dose. Comparing the treatment effects in those subjects that completed all visits (*n* = 12, Table 4) using a repeated measures ANOVA showed that none of the ozone-induced inflammation markers was significantly changed by PUR118.

**Table 3:** Composition of induced sputum (pre- vs. post-baseline ozone, *n* = 24)

		Visit 1	Visit 3
		Pre-ozone	Post-Baseline Ozone
Viability	%	85.2 (9.4)	89.3 (11.3)
TCC	10^6^/mL	1.5 (0.9)	3.8 (3.6)***
WBC	10^6^/mL	1.5 (1.0)	3.7 (3.8)***
Macrophages	% nSq	70.2 (22.6)	25.2 (21.9)***
	% WBC	72.2 (28.0)	25.5 (23.7)***
	cells/g	1.0 (0.6)	0.7 (1.0)
CD14+ Monocytes	% nSq	1.9 (1.9)	5.7 (2.4)***
	% WBC	1.9 (2.0)	5.7 (2.1)***
	cells/g	0.0 (0.1)	0.2 (0.2)***
Neutrophils	% nSq	23.6 (21.6)	64.5 (27.3)***
	% WBC	24.4 (23.5)	67.0 (24.8)***
	cells/g	0.2 (0.6)	2.2 (2.4)***
Lymphocytes	% nSq	1.5 (1.6)	0.6 (1.2)*
	%WBC	1.7 (1.5)	0.6 (1.2)*
	cells/g	0.0 (0.0)	0.0 (0.1)
Eosinophils	% nSq	0.0 (0.5)	0.3 (0.5)
	% WBC	0.0 (0.5)	0.3 (0.6)
	cells/g	0.0 (0.0)	0.0 (0.0)
Epithelial cells	% nSq	1.4 (4.9)	1.2 (1.4)
	cells/g	0.0 (0.1)	0.0 (0.0)
Squamous cells	%	13.5 (16.2)	3.8 (8.2)
IL8	pg/ml	639.6 (496.5)	1101.8 (723.1)***
IL6	pg/ml	13.1 (9.1)	39.9 (49.4)***
IL1B	pg/ml	22.8 (20.3)	36.2 (40.5)***
MMP9	ng/ml	50.9 (71.6)	152.6 (168.5)***

Median and interquartile ranges

*TCC* total cell count, *WBC* sputum white blood cells

*p < 0.05, ***p < 0.001

**Table 4:** Data for subjects that completed all visits (*n* = 12)

		SC	BL	PUR118	PUR118	PUR118
				2.8 mg dose	5.5 mg dose	11.0 mg dose
Sputum:						
Viability	%	84.5 (12.4)	85.5 (12.1)	81.1 (13.0)	82.2 (13.3)	85.0 (11.6)
TCC	10^6^/mL	1.6 (0.9)	2.7 (1.9)	3.6 (3.2)	2.2 (1.4)	3.4 (4.7)
AM	%	62.0 (23.9)	27.2 (16.4)	23.7 (22.4)	16.6 (18.6)	16.2 (21.2)
CD14 + MO	%	2.3 (1.8)	5.9 (2.2)	7.9 (4.5)	5.3 (3.6)	6.1 (3.6)
NG	%	29.5 (23.0)	63.7 (23.4)	58.1 (21.4)	71.0 (23.4)	69.3 (22.0)
LY	%	1.5 (1.6)	1.1 (1.4)	0.6 (0.4)	0.7 (0.6)	0.8 (0.6)
EO	%	0.0 (0.5)	0.3 (0.5)	0.5 (0.6)	0.5 (0.7)	0.3 (0.3)
FE	%	2.8 (5.8)	1.3 (3.0)	9.8 (11.8)	2.7 (7.7)	3.4 (5.5)
AM	10^6^/mL	0.9 (0.5)	0.6 (0.7)	0.7 (0.6)	0.4 (0.4)	0.5 (0.6)
CD14 + MO	10^6^/mL	0.0 (0.1)	0.2 (0.1)	0.2 (0.2)	0.2 (0.1)	0.2 (0.2)
NG	10^6^/mL	0.2 (0.6)	1.7 (1.9)	2.1 (2.5)	1.5 (1.3)	2.3 (3.2)
EO	10^6^/mL	0.0 (0.0)	0.0 (0.0)	0.0 (0.0)	0.0 (0.0)	0.0 (0.0)
LY	10^6^/mL	0.0 (0.0)	0.0 (0.0)	0.0 (0.0)	0.0 (0.0)	0.0 (0.0)
FE	10^6^/mL	0.1 (0.1)	0.0 (0.1)	0.2 (0.2)	0.1 (0.2)	0.1 (0.2)
Blood:						
WBC	10^6^/mL	4.5 (1.4)	8.6 (1.9)	8.2 (2.6)	8.3 (2.3)	8.4 (2.8)
NG	%	56.5 (9.5)	67.8 (9.2)	63.3 (11.2)	64.1 (4.0)	64.3 (6.1)
MO	%	10.3 (2.3)	7.8 (2.1)	8.3 (1.8)	8.3 (2.0)	7.8 (2.4)
LY	%	28.1 (11.8)	22.0 (8.6)	24.5 (10.8)	24.4 (7.4)	23.9 (5.1)
EO	%	2.1 (1.8)	1.5 (1.2)	2.2 (1.3)	1.8 (0.9)	1.7 (0.4)
BA	%	0.7 (0.3)	0.3 (0.1)	0.3 (0.1)	0.4 (0.3)	0.4 (0.2)

Median and interquartile ranges. Sputum was obtained and analysed at the screening visit (SC) and following four ozone challenges with no treatment (BL) or treatment with 2.8 mg, 5.5 mg or 11 mg doses of PUR118. Repeated measures ANOVA including BL, 2.8 mg, 5.5 mg or 11 mg doses showed no significant changes

*TCC* total cell count, *AM* macrophages, *NG* neutrophils, *LY* lymphocytes, *EO* eosinophils, *FE* bronchial epithelial cells, *MO* monocytes, *WBC* leucocytes, *BA* basophils

Treatment effects were also assessed by comparing the results of the baseline ozone exposure with the data from all subjects that received the high dose of treatment (11.0 mg PUR118, *n* = 16 subjects). In this analysis, a decrease in the percentage (*p* = 0.04) and number (*p* = 0.05) of sputum macrophages, a decrease in blood neutrophils (*p* = 0.04), and an increase in blood CD14+ cells (*p* = 0.04) compared to baseline was observed.

The white blood cell (WBC) counts and their absolute change from baseline are summarized in (Additional file 4: Table S2). Ozone-induced increases in WBCs were slightly smaller with PUR118 than salbutamol only treatment. Statistically significant differences between PUR118 doses and salbutamol only treatment for change from baseline were observed for WBCs (10^6^/mL) with the 5.5 mg PUR118 (*p* = 0.0391) and 11.0 mg PUR118 (*p* = 0.0102) dose 7 h post-ozone challenge (t-test). The ANOVA showed a significant effect for WBC (10^6^/mL) changes for time point (with the largest increase being visible at 7 h post-ozone, *p* < 0.0001), but no significant effect for dose level (*p* = 0.1781).

CD11b + neutrophil counts and their absolute change from baseline are summarized in (Additional file 5: Table S3). There were small differences between PUR118 and salbutamol only treatment for changes from baseline and no clear dose trends were observed for different PUR118 doses for all three CD11b + parameters (expressed as % total, 10^6^/mL and X-mean). Similar results were obtained for CD14+ monocytes (Additional file 6: Table S4).

Data for CRP and CC16 and their absolute changes from baseline are summarized in (Additional file 7: Table S5). Ozone-induced increases in CRP were slightly lower with PUR118 (5.5 and 11.0 mg dose) than with salbutamol only treatment with a statistically significant difference (paired t-test) between salbutamol only treatment being observed for the 5.5 mg dose at 7 h post-ozone challenge (*p* = 0.0479). The repeated measurements ANOVA showed significant effects for dose level (*p* = 0.0152) and time (*p* = 0.0022, with the highest increase visible at 24 h post-ozone: *p* = 0.0013). Differences in least squares means revealed significant differences between the 2.8 mg dose and salbutamol only treated values (*p* = 0.0028), as well as between the 5.5 mg and salbutamol only treated (*p* = 0.0359), and also between the 2.8 mg and 11.0 mg dose (*p* = 0.0360). For CC16, there were small differences in ozone-induced increases between salbutamol only treatment and different PUR118 doses without any statistically significant differences between treatments (paired t-test).

### Safety - adverse events

A total of 83 AEs were experienced by 23 (95.8 %) subjects. All but six were treatment emergent adverse events (77 TEAEs in 23 subjects, Table 5). There were no deaths or serious adverse events (SAEs). The incidence of TEAEs was similar in the PUR118 treatment periods (range 50 to 58 %) and slightly higher under pre-salbutamol only treatment (67 %). Most of the TEAEs were judged to be of mild (*n* = 50) or moderate (*n* = 26) intensity. There was only one severe TEAE (syncope) in one subject with 5.5 mg PUR118, which resolved without further treatment and was judged unrelated to treatment or study procedures. Nevertheless, the subject decided to discontinue the study. Most TEAEs were judged as unrelated to PUR118. Nine TEAEs in six subjects were judged as possibly related or related to PUR118. All TEAEs except for one mild event of abdominal hernia under 11.0 mg PUR118 treatment, had resolved by the end of study. Two TEAEs (oropharyngeal pain and upper respiratory tract infection) in one subject required PUR118 interruption during the treatment with 11.0 mg dose and 31 TEAEs in 15 subjects required drug therapy.

**Table 5:** Overview of treatment emergent adverse events (SAF, *n* = 24)

	Total			Untreated			2.8 mg dose			5.5 mg dose			11.0 mg dose		
	(*N* = 24)			(*N* = 24)			(*N* = 18)			(*N* = 19)			(*N* = 20)		
	*N* (%)^a^		n	*N* (%)^a^		n	*N* (%)^a^		n	*N* (%)^a^		n	*N* (%)^a^		n
All TEAEs	23	(95.8)	77	16	(66.7)	24	10	(55.6)	17	11	(57.9)	21	10	(50.0)	15
Severe	1	(4.2)	1	–		–	–		–	1	(5.3)	1	–		–
Possibly related	4	(16.7)	7	–		–	1	(5.6)	1	3	(15.8)	6	–		–
Related	2	(8.3)	2	–		–	–		–	1	(5.3)	1	1	(5.0)	1

*AE* adverse event, *N* number of subjects, *n* number of events, *SAF* safety analysis set, *TEAE* treatment emergent adverse event

^a^Percentages are based on the total number of subjects

No clinically significant changes in hematology and clinical chemistry parameters were observed during the study. PUR118 had no effect on FEV_1_ and FVC. The observed small differences between PUR118 and salbutamol-only treatment in FEV_1_ and FVC were not statistically significant (Additional file 8: Figure S1).

Single events of substantial changes in blood pressure (decrease or increase greater than 20 mmHg) occurred throughout the study. Up to 92 % of subjects presented with a substantial change in heart rate (decrease or increase greater than 15 bpm) 1 h, 2 h and 3 h after the ozone challenge, which was related to physical exercise. Only small changes of oral temperature and oxygen saturation were observed during the study. There were six subjects with abnormal ECG findings in continuous ECG measurements during ozone challenge. None of the findings were clinically significant.

## Discussion

With ozone challenge, expected and reproducible increases of inflammatory markers, such as sputum neutrophils and monocytes as well as IL8, IL6, and MMP9 in sputum supernatant were seen. PUR118 slightly attenuated the increase in blood total leukocytes 7 h after ozone exposure and the CRP level 24 h after ozone exposure. There were small effects on inflammatory parameters in induced sputum, but no clear dose effect. Three applications of PUR118 within the 25 h prior to an ozone challenge were not sufficient to significantly reduce ozone-induced airway inflammation. The treatment with PUR118 was safe and well tolerated.

### PUR118

PUR118 is intended for delivery to the airway surface liquid (ASL) and to the ciliated airway epithelium in the conducting airways to induce biophysical and biochemical changes to improve mucociliary clearance, reduce inflammation and reduce pathogen infection. The ASL acts as a natural barrier in the airway and consists of a mucus gel and periciliary fluid layer of approximately 8 – 13 μm and 7 – 10 μm, respectively [18]. The ASL also includes rheologically active glycoproteins as well as anti-microbial peptides that are central to host defense responses [19]. Calcium ions, as a major constituent of PUR118, interact with electronegative mucins present at the surface of the ASL. This results in conformational changes which increase the surface viscoelastic properties of the ASL. These alterations potentially lead to an enhanced barrier function of the ASL and limit pathogen ingress and infection [9–11]. PUR118 can also enhance mucociliary clearance by altering the osmotic gradient of the extracellular milieu through increased hydration of the ASL leading to thinner, and more easily clearable mucus [20, 21]. Whether an enhanced barrier function is also capable to attenuate the effects of ozone on the epithelium is unclear.

Furthermore, PUR118 is thought to modulate ion channel activity in the apical membrane of airway epithelial cells potentially leading to changes in intracellular calcium levels. Elevated intracellular calcium together with protein kinase C (PKC) activation by DAG is important for vesicle exocytosis, superoxide production by the NADPH oxidase, and JNK activation [22]. Calcium also stimulates lysosome exocytosis by activating the synaptotagmin regulator of vesicle fusion SYT7 [23]. Therefore, PUR118 may enhance innate antimicrobial response to infection from both airway epithelial cells and airway macrophages. Similarly, alterations of chemokine and cytokine gene expression could influence inflammatory cell recruitment and potentially augment phagocytic activity of macrophages. The totality of these effects may ulimately lead to a reduced risk of infection and resolution of inflammation, which may be most relevant to acute exacerbation control. However, in this study, the influence on inflammatory cell recruitment was not sufficient to significantly modulate the inflammatory response to ozone.

### Safety

The ozone challenge resulted in a small decline in FEV_1_ that ranged between 4 and 7 % from pre-exposure values, which had resolved 24 h after the challenge. The ECG findings during the ozone challenge were not of clinical significance and most likely due to the intermittent physical exercise. PUR118 treatment was safe and only mild treatment emergent adverse events were observed. Due to previous observations in studies with COPD patients, where capsules containing powder loads of 40 mg induced airway constriction in a few subjects, in this study salbutamol was inhaled before each application of PUR118. The administration of pre-dose bronchodilators is common when administering high dose dry powder formulations [24]. In order to rule out confounding by a potential anti-inflammatory effect of ß-agonists [25] the same premedication was performed at the baseline challenge.

### Design

The study was designed as an uncontrolled study, with no dry powder placebo treatment arm, as an appropriately matching dry powder placebo to the PUR118 active formulation was not available. The baseline ozone challenge with prior salbutamol administration (salbutamol) alone was therefore considered to be the appropriate control. Doses of PUR118 were selected based on the doses used in a previous clinical study (ClinicalTrials.gov Identifier: NCT01333904), which demonstrated that single and multiple doses of PUR118 up to 44 mg in healthy subjects and 22 mg with prior bronchodilation in subjects with COPD were well tolerated. In addition, the doses chosen in this study were consistent with the dose ranges found to be efficacious in a mouse model of acute ozone-induced inflammation on a milligram per kilogram body weight basis.

### Ozone challenge model

Compared to previous studies, the pre-treatment with salbutamol prior to ozone challenges did not change the extent of the inflammatory response and both sputum inflammatory markers and blood markers [12–14] were in the expected range. For the markers in blood we observed the typical increase in neutrophils, CC16, and CRP. The effects were transient and the levels nearly returned to the baseline levels at the 24 h time point. In previous ozone challenge studies it was clearly shown that the ozone induced inflammatory response can be modulated by steroids and even completely be inhibited by a CXCR2 antagonist [12, 13, 26]. As the steroid response was shown to be reproducible, a steroid treatment arm was not included in this study in order to allow the evaluation of 3 doses of PUR118. To extend an ozone challenge proof of concept study beyond a total of 4 exposures increases the duration of the study and therefore increases the risk for additional dropouts.

### Reproducibility of the ozone induced airway inflammation

The inflammatory response to ozone has been shown to be repeatable [27]. Assuming no or only a very small treatment effect after the 2.8 mg dose of PUR118 we also assessed the reproducibility of ozone induced airway inflammation by comparing the results of the baseline and 2.8 mg dose treatment visits (*n* = 16). As expected, the reproducibility for most inflammatory markers was good (Additional file 9: Table S6), underlining the suitability of the ozone challenge model for proof of concept studies and make it unlikely that a variability in ozone responsiveness contributed to the small effects in efficacy of PUR118.

### Sputum quality

The overall sputum quality in the study was good as indicated by the low median squamous cell contamination of less than 10 %. Pre-screening of subjects to include only those that are able to produce a sufficient amount and quality of sputum resulted in only 3 cases during the study in which not all measurements could be performed. The acceptability cutoff was preset to 200,000 cells as this was the minimum number needed to produce at least 2 cytospots and to perform the flow cytometric analysis. As described in Fig. 2 10 subjects were excluded that were not able to produce this number of cells. In addition 9 subjects with larger than 50 % sputum neutrophils at screening were excluded in order to assure that a sufficient increase in neutrophil percentage could be observed after ozone exposure.

### Limitations

As already mentioned we did not include a placebo group into our study as there was no adequate placebo available for PUR118 salt. In addition, the number of visits was already high for each participant, further increasing the duration of the study would have had negative effects on the recruitment and would have increased the risk of dropouts. A further limitation was that we were not able to include a validated “target engagement” biomarker, that would indicate the pharmacological activity of PUR118, but to our best knowledge, such a marker is not available and therefore we focused on the efficacy markers like airway neutrophilia in this study.

## Conclusions

Using the ozone challenge model we tested the anti-inflammatory potential of PUR118 early in the drug development process. The ozone challenge resulted in the expected and significant increase of sputum inflammatory parameters, and we observed effects on inflammatory parameters in induced sputum after treatment, however, no clear dose effect. Three applications of PUR118 within the 25 h prior to an ozone challenge were not sufficient to significantly reduce ozone-induced airway inflammation. The treatment with PUR118 was safe and well tolerated.

## Additional files

Additional file 1: Doc 1. CONSORT CHECKLIST. (DOC 218 kb) Additional file 2: Doc 2. Additional information on ethics approval and trial registration. (DOCX 15 kb) Additional file 3: Table S1. Sputum analysis. (DOCX 20 kb) Additional file 4: Table S2. White blood cells. (DOCX 16 kb) Additional file 5: Table S3. CD11b + neutrophils. (DOCX 19 kb) Additional file 6: Table S4. CD14+ monocytes. (DOCX 18 kb) Additional file 7: Table S5. CRP and CC16. (DOCX 17 kb) Additional file 8: Figure S1. Spirometry data over 26 h separately for baseline and treatment visits. Mean and standard deviation are shown for FEV1 = forced expiatory volume in 1 s (squares) and FVC = forced vital capacity (circles). (TIFF 878 kb) Additional file 9: Table S6. Correlation coefficients for selected inflammatory markers. (DOCX 15 kb)
