# Supplementary material for: Efficacy and safety of inhaled calcium lactate PUR118 in the ozone challenge model - a clinical trial
Source: BMC Pharmacol Toxicol. 2015 Aug 12;16:21. doi: 10.1186/s40360-015-0021-1 (PMC4533952; doi:10.1186/s40360-015-0021-1)
Supplement: Additional file 2: Doc 2. — Additional information on ethics approval and trial registration. (DOCX 15 kb) [file 40360_2015_21_MOESM2_ESM.docx]

**Supporting Information Ethics/Registration**

The study was approved by the Ethical Committee of Hannover Medical School on March 30^th^ 2012. The respective approval of the competent authority (Bundesinstitut für Arzneimittel und Medizinprodukte, BfArM) was received 11^th^ June 2012. The study protocol is dated from the 19^th^ of June. Here we changed the dose application sequence due to difficulties in the production process of the inhalation capsules. This version of the study protocol, was send to the ethics committee on June 25^th^ and approved June 27^th^, 2 days after including the first patient into the study. BfArM approved this version of the protocol on July 16^th^.

The inclusion of the first subject on June 25^th^ 2012 was based on the ethics approval from March 30^th^ 2012, as the amendment to the study protocol submitted June 25^th^ only concerned the dosing phase of the study. This amendment had no effects on the screening phase of the study which started with testing of inclusion criteria. The subjects included prior to the 27^th^ of June all provided an additional written informed consent in which the changes set into place on June 27^th^ were also acknowledged. All subjects included prior to June 27^th^ were rated as screening failures. The first dosing occurred on August 6^th^.

Due to a difficult recruitment phase during the summer month June and July 2012 we asked the ethics committee on July 30^th^ to change the text for newspaper advertisement, which are used to recruit the volunteers. This change was approved on the 9^th^ of August.

As the study sponsor, CRO and clinical site anticipated the above amendments to the study protocol, registration on a publicly accessible database was delayed until the final approvals were in place to maintain clarity. In addition, due to different views between the US Sponsor of the trial and the European CRO and the Fraunhofer study centre on the applicable laws concerning the requirements for registration of this clinical trial, the registration under the ClinicalTrials.gov Identifier: NCT01690949 was delayed until September 12^th^, 2012.

In this respect the authors confirm that all ongoing and related trials for this drug/intervention are registered.
