# Supplementary material for: Efficacy and safety of inhaled calcium lactate PUR118 in the ozone challenge model - a clinical trial
Source: BMC Pharmacol Toxicol. 2015 Aug 12;16:21. doi: 10.1186/s40360-015-0021-1 (PMC4533952; doi:10.1186/s40360-015-0021-1)
Supplement: Additional file 3: Table S1. — Sputum analysis. (DOCX 20 kb) [file 40360_2015_21_MOESM3_ESM.docx]

| Table S1: Sputum analysis (SAF, N = 24) | | | | | | | | | | | |
| --- | --- | --- | --- | --- | --- | --- | --- | --- | --- | --- | --- |
|  |  | |  | **Absolute change from Baseline** | | | | | | |  |
|  | **Baseline (N = 24)** | | **2.8 mg Dose (N = 16)^a^** | | | **5.5 mg Dose (N = 18)** | | | **11.0 mg Dose (N = 18)** | | |
| **Parameter** | Median (range) | | Median (range) | | | Median (range) | | | Median (range) | | |
| Weight of spu. (g) | 1.0 | (0.3-4.8) | na | |  | na |  | | na |  | |
| Weight of filtrate (g) | 8.2 | (2.2-39.9) | na | |  | na |  | | na |  | |
| Weight of resuspension (g) | 1.1 | (0.4-3.3) | na | |  | na |  | | na |  | |
| Viability of spu. cells (%) | 89.3 | (67.5-98.2) | na | |  | na |  | | na |  | |
| Total n-sq. cells (10^6^/g spu.) | 3.8 | (0.8-11.2) | -0.3 | | (-2.7-3.5) | 0.3 | (-3.9-3.6) | | 0.5 | (-3.1-4.9) | |
| Total leukocytes (10^6^/g spu.) | 3.7 | (0.8-11.2) | -0.4 | | (-2.7-3.4) | 0.3 | (-3.9-3.7) | | 0.5 | (-3.2-5.0) | |
| Macrophages (% n-sq. cells) | 25.2 | (2.2-54.6) | -3.3 | | (-22.4-36.5) | -3.8 | (-23.4-9.6) | | -6.3 | (-43.7-9.8) | |
| Macrophages (% leukocytes) | 25.5 | (2.2-59.6) | -2.9 | | (-20.9-40.4) | -5.6 | (-22.9-14.9) | | -6.3 | (-48.7-11.5) | |
| Macrophages (10^6^/g spu.) | 0.7 | (0.1-2.5) | <-0.1 | | (-1.1-0.6) | -0.1 | (-1.3-0.5) | <-0.1 | | (-0.6-1.2) | |
| CD14^+^ Mono. (% n-sq. cells) | 5.7 | (1.9-10.5) | 1.3 | | (-2.6-4.0) | 0.5 | (-1.9-2.8) | | 0.6 | (-3.6-8.6) | |
| CD14^+^ Mono. (% leukocytes) | 5.7 | (1.9-10.6) | 1.6 | | (-2.5-5.4) | 0.5 | (-1.7-2.5) | | 0.9 | (-3.5-8.3) | |
| CD14^+^ Mono. (10^6^/g spu.) | 0.2 | (<0.1-0.9) | <0.1 | | (-0.3-0.4) | <0.1 | (-0.1-0.5) | | 0.1 | (-0.2-0.4) | |
| Neutrophils (% n-sq. cells) | 64.5 | (32.0-90.6) | -4.7 | | (-37.5-19.7) | 5.3 | (-19.3-23.1) | | 6.9 | (-25.2-48.5) | |
| Neutrophils (% leukocytes) | 67.0 | (34.9-90.6) | 0.8 | | (-36.8-20.9) | 4.4 | (-16.1-21.7) | | 6.4 | (-14.2-46.0) | |
| Neutrophils (10^6^/g spu.) | 2.2 | (0.3-10.1) | -0.2 | | (-2.7-3.8) | 0.2 | (-4.2-3.2) | | 0.4 | (-2.6-4.9) | |
| Lymphocytes (% n-sq. cells) | 0.6 | (0.0-3.5) | -0.1 | | (-1.7-2.6) | <-0.1 | (-1.8-2.3) | | -0.3 | (-1.3-1.4) | |
| Lymphocytes (% leukocytes) | 0.6 | (0.0-3.5) | <0.1 | | (-1.7-2.6) | -0.1 | (-1.8-2.7) | | -0.3 | (-1.2-1.4) | |
| Lymphocytes (10^6^/g spu.) | <0.1 | (0.0-0.1) | 0.0 | | (-0.1-0.1) | <-0.1 | (-0.1-0.1) | | <0.1 | (-0.1-0.1) | |
| Eosinophils (% n-sq. cells) | 0.3 | (0.0-1.6) | 0.1 | | (-1.5-1.2) | 0.2 | (-1.5-1.4) | | <0.1 | (-1.6-1.0) | |
| Eosinophils (% leukocytes) | 0.3 | (0.0-1.6) | 0.1 | | (-1.5-1.4) | 0.3 | (-1.5-1.4) | | 0.1 | (-1.6-1.1) | |
| Eosinophils (10^6^/g spu.) | <0.1 | (0.0-0.1) | 0.0 | | (-0.1-<0.1) | <0.1 | (-0.1-0.1) | | <0.1 | (-0.1-<0.1) | |
| Br. epith. cells (% n-sq. cells) | 1.2 | (0.0-10.0) | 2.9 | | (-8.9-15.6) | 0.8 | (-10.0-22.6) | | -0.3 | (-7.9-30.1) | |
| Br. epith. cells (10^6^/g spu.) | <0.1 | (0.0-0.3) | <0.1 | | (-0.2-0.6) | <0.1 | (-0.2-0.5) | <-0.1 | | (-0.2-0.3) | |
| Squa. cells (%) | 3.8 | (0.8-32.0) | 2.3 | | (-14.2-16.7) | 1.0 | (-7.4-11.2) | | 1.6 | (-18.2-29.6) | |
| Interleukine-8 (10^3^pg/mL) | 1.1 | (0.3-3.1) | <-0.1 | | (-1.4-1.0) | 0.2 | (-1.5-1.2) | | 0.2 | (-0.7-2.0) | |
| Interleukine-6(pg/mL) | 39.9 | (11.4-91.7) | 0.2 | | (-73.4-40.4) | -3.1 | (-31.6-100.3) | | -2.1 | (-57.9-64.7) | |
| Interleukine-1b (pg/mL) | 36.2 | (15.7-194.9) | -0.5 | | (-122.5-26.7) | 1.4 | (-81.7-50.4) | <-0.1 | | (-97.9-81.9) | |
| MMP-9 (ng/mL) | 152.6 | (31.0-606.3) | -12.9 | | (-238.8-313.7) | 48.3 | (-277.2-273.9) | | 24.9 | (-176.4-381.7) | |
| Baseline for each dose level was the 6 h post-ozone measurement on Visit 3.  <0.1 equals values between 0.0 and 0.05, <-0.1 equals values between -0.001 and -0.05.  ^a^ N = 17 for IL8, IL6, IL1B and MMP9 measurements.  Br. epith. = bronchial epithelial cells, N = number of subjects, na = not assessed, n-sq. = non-squamous, spu. = sputum, Mono. = monocytes, MMP9 = matrix metalloprotease 9, SAF = safety analysis set, Squa. cells =  Squamous cells. | | | | | | | | | | | |
