# Supplementary material for: Efficacy and safety of inhaled calcium lactate PUR118 in the ozone challenge model - a clinical trial
Source: BMC Pharmacol Toxicol. 2015 Aug 12;16:21. doi: 10.1186/s40360-015-0021-1 (PMC4533952; doi:10.1186/s40360-015-0021-1)
Supplement: Additional file 4: Table S2. — White blood cells. (DOCX 16 kb) [file 40360_2015_21_MOESM4_ESM.docx]

| Table S2: White blood cells (SAF, N = 24) | | | | | | | | | | | | | | | |  |  |
| --- | --- | --- | --- | --- | --- | --- | --- | --- | --- | --- | --- | --- | --- | --- | --- | --- | --- |
|  |  | | | | |  | | | | | | **Absolute change from Baseline** | | | | |  |
|  |  | | | | |  | **WBC (10^6^/mL)** | | |  | | **WBC (10^6^/mL)** | | | | |  |
| **Dose** |  |  | | | | Median (range) | | | | | | Median(range) | | | | |  |
| **Untreated (N = 24)** | | | | | |  | |  | | |  | | |  | |  |  |
| Baseline | | | | | 5.1 | | | | (2.5-10.5) | | | | N/A | |  | | |
| 7 h post-ozone | | | | | 9.4 | | | | (6.4-17.8) | | | | 4.3 | | (1.6-11.7) | | |
| 24 h post-ozone | | | | | 5.1 | | | | (2.7-9.6) | | | | 0.1 | | (-1.2-5.3) | | |
| **2.8 mg (N = 18)** | | | | |  | | | |  | | | |  | |  | | |
| Baseline | | | |  | 5.3 | | | | (3.2-10.0) | | | | N/A | |  | | |
| 7 h post-ozone | | | | | 9.8 | | | | (6.0-15.6) | | | | 3.5 | | (0.6-9.6) | | |
| 24 h post-ozone | | | | | 5.3 | | | | (3.0-8.9) | | | | 0.1 | | (-2.4-1.6) | | |
| **5.5 mg (N = 19)** | | | | |  | | | |  | | | |  | |  | | |
| Baseline | | |  | | 4.8 | | | | (3.7-7.6) | | | | N/A | |  | | |
| 7 h post-ozone | | | | | 9.1 | | | | (5.8-14.2) | | | | 4.0 | | (1.5-6.6)* | | |
| 24 h post-ozone | | | | | 4.8 | | | | (3.0-7.2) | | | | -0.1 | | (-2.3-0.9) | | |
| **11 mg (N = 20)** | | | | |  | | | |  | | | |  | |  | | |
| Baseline | | |  | | 5.6 | | | | (3.3-9.9) | | | | N/A | |  | | |
| 7 h post-ozone | | | | | 10.1 | | | | (6.2-12.8) | | | | 3.9 | | (0.9-6.9)* | | |
| 24 h postozone | | | | | 5.4 | | | | (2.7-8.2) | | | | -0.2 | | (-2.9-1.8) | | |
| Baseline for each dose level was the pre-salbutamol measurement on the Day 1 of each treatment period.  * p<0.05 for difference vs salbutamol only treatment (paired t-test).  N = number of subjects, N/A = not applicable, SAF = safety analysis set, WBC = white blood cells. | | | | | | | | | | | | | | | |  |  |
