# Supplementary material for: Efficacy and safety of inhaled calcium lactate PUR118 in the ozone challenge model - a clinical trial
Source: BMC Pharmacol Toxicol. 2015 Aug 12;16:21. doi: 10.1186/s40360-015-0021-1 (PMC4533952; doi:10.1186/s40360-015-0021-1)
Supplement: Additional file 5: Table S3. — CD11b + neutrophils. (DOCX 19 kb) [file 40360_2015_21_MOESM5_ESM.docx]

| Table S3: CD11b+ neutrophils (SAF, N = 24) | | | | | | | | | | | | | | | | | | | | | | | | | | | | | | |
| --- | --- | --- | --- | --- | --- | --- | --- | --- | --- | --- | --- | --- | --- | --- | --- | --- | --- | --- | --- | --- | --- | --- | --- | --- | --- | --- | --- | --- | --- | --- |
|  | |  | | |  | | | | | |  | | | | | **Absolute change from Baseline** | | | | | | | | | | | | | | |
|  | | **CD11b^+^ Neutr. (% total)** | | | **CD11b^+^ Neutr. (10^6^/mL)** | | | | | | **CD11b^+^ Neutr. (X-mean)** | | | | | **CD11b^+^ Neutr. (% total)** | | | | | **CD11b^+^ Neutr. (10^6^/mL)** | | | | | **CD11b^+^ Neutr. (X-mean)** | | | | |
| **Dose** | | Median (range) | | | Median (range) | | | | | | Median (range) | | | | | Median (range) | | | | | Median (range) | | | | | Median (range) | | | | |
| **Untreated (N = 24)** | | | | |  | | |  | |  | | |  | |  | | |  | |  | | |  | |  | | |  | |  |
| Baseline | | 56.3 | | (36.8-72.6) | | | 2.6 | | (1.1-6.7) | | 4.7 | | | (1.1-17.1) | | N/A | | |  | | N/A | | |  | | N/A | | |  | |
| 7h post-o | | 73.3 | | (51.5-82.7) | | | 6.7 | | (4.7-14.7) | | 4.0 | | | (-1.6-15.9) | | 14.1 | | | (-1.9-40.7) | | 3.9 | | | (1.5-11.2) | | -1.1 | | | (-6.6-6.8) | |
| 24h post-o | | 61.2 | | (45.6-81.5) | | | 3.1 | | (1.5-7.5) | | 3.2 | | | (-1.5-13.9) | | 3.9 | | | (-2.8-36.7) | | 0.4 | | | (-0.9-5.7) | | -1.9 | | | (-7.0-6.2) | |
| **2.8 mg (N = 18)** | | | | | | | | |  | |  | | |  | |  | | |  | |  | | |  | |  | | |  | |
| Baseline | | 56.0 | | (37.8-82.4) | | | 2.8 | | (1.7-6.7) | | 2.6 | | | (-1.7-9.3) | | N/A | | |  | | N/A | | |  | | N/A | | |  | |
| 7h post-o | | 71.3 | | (54.5-82.6) | | | 7.2 | | (4.0-11.8) | | 7.3 | | | (-0.7-16.9) | | 13.4 | | | (-10.2-28.3) | | 2.7 | | | (1.1-8.9) | | 1.4 | | | (-1.2-18.6) | |
| 24h post-o | | 64.0 | | (54.0-81.4) | | | 3.5 | | (1.6-6.4) | | 2.5 | | | (-1.5-14.6) | | 3.4 | | | (-15.6-25.8) | | 0.5 | | | (-2.8-2.0) | | 0.3 | | | (-4.4-16.3) | |
| **5.5 mg (N = 19)** | | | | | | | | |  | |  | | |  | |  | | |  | |  | | |  | |  | | |  | |
| Baseline | | 57.8 | | (39.9-70.1) | | | 2.9 | | (1.5-4.4) | | 2.3 | | | (<-0.1-17.6) | | N/A | | |  | | N/A | | |  | | N/A | | |  | |
| 7h post-o | | 72.8 | | (57.3-79.2) | | | 6.6 | | (4.2-10.3) | | 5.4 | | | (0.2-19.0) | | 14.0 | | | (0.4-31.3) | | 3.1 | | | (1.2-6.0)* | | 1.2 | | | (-6.5-10.4) | |
| 24h post-o | | 60.9 | | (46.1-76.7) | | | 3.0 | | (1.4-5.3) | | 1.8 | | | (<0.1-14.1) | | 3.1 | | | (-9.1-15.5) | | <0.1 | | | (-1.6-1.3) | | -0.6 | | | (-7.3-7.2) | |
| **11 mg (N = 20)** | | | | | | | | |  | |  | | |  | |  | | |  | |  | | |  | |  | | |  | |
| Baseline | | 59.3 | | (42.7-73.3) | | | 3.2 | | (1.4-5.8) | | 3.3 | | | (0.7-17.6) | | N/A | | |  | | N/A | | |  | | N/A | | |  | |
| 7h post-o | | 71.6 | | (52.8-81.1) | | | 7.0 | | (3.6-9.4) | | 5.1 | | | (0.4-18.6) | | 11.1 | | | (1.0-22.2) | | 3.5 | | | (1.2-5.4)* | | 1.0 | | | (-8.2-9.3) | |
| 24h post-o | | 61.1 | | (41.1-75.2) | | | 3.3 | | (1.4-5.4) | | 3.0 | | | (-4.1-12.1) | | 1.5 | | | (-9.2-15.3) | | -0.1 | | | (-1.5-1.6)* | | -0.6 | | | (-10.5-9.0) | |
| Baseline for each dose level was the pre-salbutamol measurement on the Day 1 of each treatment period.  <0.1 equals values between 0.0 and 0.05, <-0.1 equals values between -0.001 and -0.05.  * p<0.05 for difference vs salbutamol only treatment from Baseline (paired t-test).  N = number of subjects, N/A = not applicable, Neutr. = neutrophils, post-o = post-ozone challenge, SAF = safety analysis set, X‑mean = mean fluorescence intensity. | | | | | | | | | | | | | | | | | | | | | | | | | | | | | | |
