# Supplementary material for: Efficacy and safety of inhaled calcium lactate PUR118 in the ozone challenge model - a clinical trial
Source: BMC Pharmacol Toxicol. 2015 Aug 12;16:21. doi: 10.1186/s40360-015-0021-1 (PMC4533952; doi:10.1186/s40360-015-0021-1)
Supplement: Additional file 6: Table S4. — CD14+ monocytes. (DOCX 18 kb) [file 40360_2015_21_MOESM6_ESM.docx]

| Table S4: CD14+ monocytes (SAF, N = 24) | | | | | | | | | | | | | | | | | | | | |
| --- | --- | --- | --- | --- | --- | --- | --- | --- | --- | --- | --- | --- | --- | --- | --- | --- | --- | --- | --- | --- |
|  | |  | | |  | | |  | | | **Absolute change from Baseline** | | | | | | | | | |
|  | | **CD14^+^ Mono. (% total)** | | | **CD14^+^ Mono. (10^6^/mL)** | | | **CD14^+^ Mono. (X-mean)** | | | **CD14^+^ Mono. (% total)** | | | | **CD14^+^ Mono. (10^6^/mL)** | | | **CD14^+^ Mono. (X-mean)** | | |
| **Dose** | | Median (range) | | | Median (range) | | | Median (range) | | | Median (range) | | | | Median (range) | | | Median (range) | | |
| **Untreated (N = 24)** | | | | |  | |  |  | |  |  | |  | |  | |  |  | |  |
| Baseline | | 7.3 | | (5.0-12.9) | 0.4 | | (0.2-0.6) | 57.6 | | (44.7-81.7) | N/A | |  | | N/A | |  | N/A | |  |
| 7 h post-o | | 6.1 | | (3.3-9.1) | 0.5 | | (0.3-1.0) | 57.0 | | (45.3-69.6) | -1.9 | | (-4.7-1.1) | | 0.2 | | (-0.1-0.5) | 0.5 | | (-21.7-10.7) |
| 24 h post-o | | 7.3 | | (4.5-10.9) | 0.4 | | (0.1-0.8) | 55.1 | | (41.5-71.6) | -0.1 | | (-3.4-3-1) | | <-0.1 | | (-0.3-0.2) | -3.0 | | (-23.8-8.9) |
| **2.8 mg (N = 18)** | | | | | | |  |  | |  |  | |  | |  | |  |  | |  |
| Baseline | | 7.6 | | (5.4-13.6) | 0.4 | | (0.2-0.7) | 57.1 | | (47.7-69.1) | N/A | |  | | N/A | |  | N/A | |  |
| 7 h post-o | | 6.3 | | (3.9-9.5) | 0.5 | | (0.3-1.0) | 55.8 | | (47.6-73.9) | -1.6 | | (-4.1-0.8) | | 0.2 | | (-0.1-0.5) | 0.9 | | (-9.8-15.9) |
| 24 h post-o | | 7.0 | | (3.6-14.1) | 0.4 | | (0.2-0.7) | 56.5 | | (39.9-63.3) | -0.1 | | (-2.2-1.7) | <-0.1 | | | (-0.2-0.2) | -2.3 | | (-8.6-6.8) |
| **5.5 mg (N = 19)** | | | | | | |  |  | |  |  | |  | |  | |  |  | |  |
| Baseline | | 7.4 | | (6.2-11.9) | 0.4 | | (0.3-0.5) | 57.0 | | (46.0-71.0) | N/A | |  | | N/A | |  | N/A | |  |
| 7 h post-o | | 5.9 | | (4.0-8.9) | 0.6 | | (0.3-0.8) | 55.4 | | (1.3-66.9) | -1.6 | | (-3.4- -0.4) | | 0.2 | | (<-0.1-0.3) | -1.5 | | (-69.1-12.5) |
| 24 h post-o | | 7.7 | | (5.8-11.5) | 0.4 | | (0.2-0.6) | 53.3 | | (45.4-69.2) | -0.3 | | (-2.2-1.5) | | <-0.1 | | (-0.2-0.1) | -2.6 | | (-13.3-11.2) |
| **11.0 mg (N = 20)** | | | | | | |  |  | |  |  | |  | |  | |  |  | |  |
| Baseline | | 7.7 | | (5.0-13.2) | 0.4 | | (0.3-0.7) | 55.5 | | (44.3-72.0) | N/A | |  | | N/A | |  | N/A | |  |
| 7 h post-o | | 6.4 | | (4.4-10.1) | 0.6 | | (0.4-1.1) | 58.6 | | (43.2-74.1) | -1.5 | | (-3.4-0.1) | | 0.2 | | (<-0.1-0.4) | 2.6 | | (-19.4-17.7) |
| 24 h post-o | | 7.4 | | (4.4-11.7) | 0.4 | | (0.2-0.6) | 56.1 | | (43.4-64.4) | -0.5 | | (-1.8-2.3) | | <-0.1 | | (-0.2-0.1) | -1.5 | | (-12.1-7.2) |
| Baseline for each dose level was the pre-salbutamol measurement on the Day 1 of each treatment period.  <0.1 equals values between 0.0 and 0.05, <-0.1 equals values between -0.001 and -0.05.  N = number of subjects, Mono. = monocytes, post-o = post-ozone challenge, SAF = safety analysis set, X‑mean = mean fluorescence intensity. | | | | | | | | | | | | | | | | | | | | |
