# Supplementary material for: Efficacy and safety of inhaled calcium lactate PUR118 in the ozone challenge model - a clinical trial
Source: BMC Pharmacol Toxicol. 2015 Aug 12;16:21. doi: 10.1186/s40360-015-0021-1 (PMC4533952; doi:10.1186/s40360-015-0021-1)
Supplement: Additional file 7: Table S5. — CRP and CC16. (DOCX 17 kb) [file 40360_2015_21_MOESM7_ESM.docx]

| Table S5: CRP and CC16 (SAF, N = 24) | | | | | | | | | | |
| --- | --- | --- | --- | --- | --- | --- | --- | --- | --- | --- |
|  |  | |  | |  | **Absolute change from Baseline** | | | |  |
|  | **CRP (mg/L)** | | **CC16 (ng/mL)** | | **CRP (mg/L)** | | | **CC16 (ng/mL)** | | |
| **Dose** | Median (range) | | Median (range) | | Median (range) | | | Median (range) | | |
| **Untreated (N = 24)** |  |  |  |  |  | |  |  |  | |
| Baseline | 1.4 | (0.5-21.4) | 7.3 | (2.7-10.4) | N/A | |  | N/A |  | |
| 7 h post-dose | 1.2 | (0.5-12.9) | 10.9 | (4.7-20.6) | -0.3 | | (-8.5-1.0) | 3.7 | (-3.9-14.4) | |
| 24 h post-dose | 1.8 | (0.5-12.1) | 6.9 | (3.7-10.6) | 0.2 | | (-11.9-9.0) | -0.6 | (-3.8-3.4) | |
| **2.8 mg (N = 18)** |  |  |  |  |  | |  |  |  | |
| Baseline | 1.3 | (0.5-4.9) | 7.7 | (5.5-12.7) | N/A | |  | N/A |  | |
| 7 h post-dose | 1.4 | (0.5-21.3) | 10.8 | (4.9-17.6) | 0.1 | | (-0.9-18.8) | 2.3 | (-1.7-7.8) | |
| 24 h post-dose | 1.5 | (0.5-23.1) | 6.5 | (4.3-11.6) | 0.3 | | (-1.3-20.6) | -0.4 | (-4.5-3.1) | |
| **5.5 mg (N = 19)** |  |  |  |  |  | |  |  |  | |
| Baseline | 1.1 | (0.5-6.5) | 7.1 | (4.9-11.2) | N/A | |  | N/A |  | |
| 7 h post-dose | 1.1 | (0.5-8.5) | 11.0 | (6.3-18.0) | 0.2 | | (-1.0-2.0) | 4.6 | (<-0.1-9.2) | |
| 24 h post-dose | 1.5 | (0.5-11.9) | 6.5 | (4.1-9.3) | 0.6 | | (-0.6-5.4) | -0.3 | (-4.7-1.8) | |
| **11.0 mg (N = 20)** |  |  |  |  |  | |  |  |  | |
| Baseline | 1.3 | (0.5-2.5) | 6.9 | (3.4-11.2) | N/A | |  | N/A |  | |
| 7 h post-dose | 1.0 | (0.5-2.3) | 11.2 | (6.7-20.2) | -0.1 | | (-1.4-0.6) | 3.9 | (0.9-9.0) | |
| 24 h post-dose | 1.4 | (0.5-6.6) | 7.1 | (4.2-12.2) | 0.3 | | (-0.7-4.9) | -0.3 | (-2.3-6.4) | |
| Baseline for each dose level was the pre-salbutamol measurement on the Day 1 of each treatment period.  <0.1 equals values between 0.0 and 0.05, <-0.1 equals values between -0.001 and -0.05.  CRP = C-reactive protein, CC16 = clara cell protein 16, N = number of subjects, SAF = safety analysis set. | | | | | | | | | | |
