# Supplementary figures and images for: Efficacy and safety of inhaled calcium lactate PUR118 in the ozone challenge model - a clinical trial
Source: BMC Pharmacol Toxicol. 2015 Aug 12;16:21. doi: 10.1186/s40360-015-0021-1 (PMC4533952; doi:10.1186/s40360-015-0021-1)

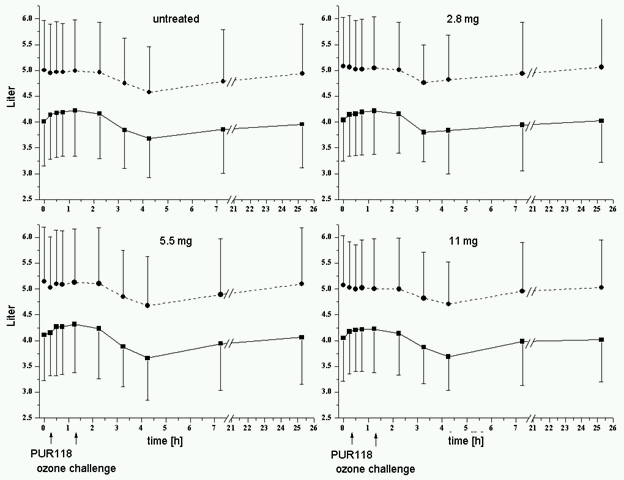

Supplement: Additional file 8: Figure S1. — Spirometry data over 26 h separately for baseline and treatment visits. Mean and standard deviation are shown for FEV1 = forced expiatory volume in 1 s (squares) and FVC = forced vital capacity (circles). (TIFF 878 kb) [file 40360_2015_21_MOESM8_ESM.tiff]
