# Supplementary material for: Efficacy and safety of inhaled calcium lactate PUR118 in the ozone challenge model - a clinical trial
Source: BMC Pharmacol Toxicol. 2015 Aug 12;16:21. doi: 10.1186/s40360-015-0021-1 (PMC4533952; doi:10.1186/s40360-015-0021-1)
Supplement: Additional file 9: Table S6. — Correlation coefficients for selected inflammatory markers. (DOCX 15 kb) [file 40360_2015_21_MOESM9_ESM.docx]

**Table S6:** Correlation coefficients for selected inflammatory markers showing the reproducibility of the ozone challenge model

|  | **BL vs. low dose** | **BL vs. med. dose** |
| --- | --- | --- |
|  | n=16 | n=18 |
|  |  |  |
| AM (%) | 0.69*** | 0.76*** |
| NG (%) | 0.71*** | 0.77*** |
| MO(%) | 0.75*** | 0.91*** |
| TCC(10^6^/mL) | 0.57* | 0.78*** |
| AM (10^6^/mL) | 0.67*** | 0.51* |
| NG (10^6^/mL) | 0.67*** | 0.76*** |
| MO (10^6^/mL) | 0.45 | 0.80*** |
| IL8 (pg/mL) | 0.59* | 0.49* |
| MMP9 (ng/mL) | 0.54* | 0.69*** |
|  |  |  |
| TCC: total cell count, AM: macrophages, NG: neutrophils, MO: monocytes, IL8: interleukin 8, MMP9: matrix metalloprotease 9, BL: baseline, med: medium  * p<0.05, *** p<0.005 | | |
